# Supplementary material for: Predicting future community-level ocular Chlamydia trachomatis infection prevalence using serological, clinical, molecular, and geospatial data
Source: PLoS Negl Trop Dis. 2022 Mar 11;16(3):e0010273. doi: 10.1371/journal.pntd.0010273 (PMC8942265; doi:10.1371/journal.pntd.0010273)
Supplement: S1 Table — (DOCX) [file pntd.0010273.s001.docx]

## S1 Table. Description and sources of geospatial variables explored for prediction analysis.

| **Variable** | **Description** | **Temporal resolution** | | **Source** |
| --- | --- | --- | --- | --- |
| ***Environmental variables*** | | | | |
| Precipitation | Precipitation (mm) | Daily | CHIRPS [1] | |
| Maximum temperature | Maximum temperature (°C) | Monthly | TerraClimate [2] | |
| Minimum temperature | Minimum temperature (°C) | Monthly | TerraClimate [2] | |
| EVI | Enhanced vegetation index | 16-day period | MODIS [3] | |
| Elevation | Average digital elevation (km) | Static, 2000 | SRTM [4] | |
| Slope | Derived terrain slope (degrees) | Static, 2000 | SRTM [4] | |
| Surface water | Landsat-derived presence/absence of surface water at any time during the month | Monthly | Joint Research Center Global Surface Water [5] | |
| ***Demographic variables*** | | |  | |
| Population | Overall population density | Static, 2018 | HRSL [6] | |
| Age distribution | 0-5 years old, proportion of population | Static, 2019 | HRSL [6] | |
| Sex distribution | Female, proportion of population | Static, 2019 | HRSL [6] | |
| ***Socioeconomic variables*** | | |  | |
| Night lights | Radiance (nanoWatts/cm2/sr) excluding negative values and pixels based on 5 or fewer cloud-free observations | Monthly | VIIRS [7] | |
| Distance to roads | Euclidean distance from center of grid cell to nearest road (km) | Static, 2020 | OpenStreetMap [8] | |
| Access to care | Land-based travel time to nearest healthcare facility (minutes), all transport and non-motorized transport only (i.e. walking-only) | Static, 2019 | Malaria Atlas Project [9] | |

**REFERENCES**

1. Funk C, Peterson P, Landsfeld M, Pedreros D, Verdin J, Shukla S, et al. The climate hazards infrared precipitation with stations—a new environmental record for monitoring extremes. Sci Data. 2015 Dec;2(1):150066.

2. Abatzoglou JT, Dobrowski SZ, Parks SA, Hegewisch KC. TerraClimate, a high-resolution global dataset of monthly climate and climatic water balance from 1958–2015. Sci Data. 2018 Dec;5(1):170191.

3. Didan K. MOD13Q1 MODIS/Terra Vegetation Indices 16-Day L3 Global 250m SIN Grid V006 [Data set] [Internet]. NASA EOSDIS Land Processes DAAC; 2015. Available from: https://doi.org/10.5067/MODIS/MOD13Q1.006

4. Jarvis A, Reuter H, Nelson A, Guevara E. Hole-filled SRTM for the globe Version 4, available from the CGIAR-CSI SRTM 90m [Internet]. 2008. Available from: http://srtm.csi.cgiar.org

5. Pekel J-F, Cottam A, Gorelick N, Belward AS. High-resolution mapping of global surface water and its long-term changes. Nature. 2016 Dec;540(7633):418–22.

6. Tiecke TG, Liu X, Zhang A, Gros A, Li N, Yetman G, et al. Mapping the world population one building at a time. ArXiv171205839 Cs [Internet]. 2017 Dec 15 [cited 2020 Oct 1]; Available from: http://arxiv.org/abs/1712.05839

7. Elvidge CD, Baugh K, Zhizhin M, Hsu FC, Ghosh T. VIIRS night-time lights. Int J Remote Sens. 2017 Nov 2;38(21):5860–79.

8. OpenStreetMap contributors. Planet dump retrieved from https://planet.osm.org. 2017.

9. Weiss DJ, Nelson A, Vargas-Ruiz CA, Gligorić K, Bavadekar S, Gabrilovich E, et al. Global maps of travel time to healthcare facilities. Nat Med [Internet]. 2020 Sep 28 [cited 2020 Nov 18]; Available from: http://www.nature.com/articles/s41591-020-1059-1
