# Supplementary material for: Predicting future community-level ocular Chlamydia trachomatis infection prevalence using serological, clinical, molecular, and geospatial data
Source: PLoS Negl Trop Dis. 2022 Mar 11;16(3):e0010273. doi: 10.1371/journal.pntd.0010273 (PMC8942265; doi:10.1371/journal.pntd.0010273)
Supplement: S2 Table — (DOCX) [file pntd.0010273.s002.docx]

## S2 Table. Number of children evaluated across 40 study communities by trachoma indicator, age group and study month.

| Month | **Number evaluated for indicator, 0–5-year-olds** | | | |  | **Number evaluated for indicator, 6–9-year-olds** | | | |
| --- | --- | --- | --- | --- | --- | --- | --- | --- | --- |
|  | Overall | PCR^1^ | TF/TI^2^ | Serology |  | Overall | PCR^1^ | TF/TI^2^ | Serology^3^ |
| 0 | 1,269 | 1,258 | 1,256 | 1,245 |  | 1,135 | 1,129 | 1,085 | 1,109 |
| 12 | 1,162 | 1,154 | 1,151 | 1,122 |  | 1,092 | 1,090 | 1,072 | 0 |
| 24 | 1,214 | 1,210 | 1,206 | 1,200 |  | 1,208 | 1,206 | 1,204 | 0 |
| 36 | 1,192 | 1,183 | 1,181 | 1,188 |  | 1,218 | 1,212 | 1,193 | 1,214 |

*1 Polymerase chain reaction*

*2 Trachomatous inflammation—follicular / trachomatous inflammation—intense*

*3 Serology was not measured for a random sample of 6–9-year-olds at months 12 and 24*
