# Supplementary material for: Predicting future community-level ocular Chlamydia trachomatis infection prevalence using serological, clinical, molecular, and geospatial data
Source: PLoS Negl Trop Dis. 2022 Mar 11;16(3):e0010273. doi: 10.1371/journal.pntd.0010273 (PMC8942265; doi:10.1371/journal.pntd.0010273)
Supplement: S3 Table — (DOCX) [file pntd.0010273.s003.docx]

## S3 Table. Community-level seroprevalence across 40 study communities by antigen, age group, and study month.

| Month | **Median prevalence (%), 0–5-year-olds (IQR)** | | | |  | **Median prevalence (%), 6–9-year-olds (IQR)^3^** | | | |
| --- | --- | --- | --- | --- | --- | --- | --- | --- | --- |
|  | n^1^ | Serology^2^ | Pgp3 | CT694 |  | n^1^ | Serology^2^ | Pgp3 | CT694 |
| 0 | 1,245 | 25.0 (10.1-34.8) | 30.4 (13.3-40.7) | 26.5 (10.1-34.9) |  | 1,109 | 49.2 (29.8-60.2) | 60.0 (39.4-70.6) | 49.2 (32.1-61.5) |
| 12 | 1,122 | 29.7 (15.6-40.2) | 36.0 (21.8-47.5) | 30.7 (15.6-46.8) |  | 0 | - | - | - |
| 24 | 1,200 | 33.3 (20.5-39.0) | 38.2 (24.0-45.8) | 33.3 (20.5-39.7) |  | 0 | - | - | - |
| 36 | 1,188 | 33.3 (23.5-42.3) | 40.0 (32.5-52.2) | 33.3 (24.6-42.3) |  | 1,214 | 50.8 (28.9-65.4) | 58.7 (34.6-77.5) | 50.8 (28.9-65.4) |

*1 Number of children tested for serology*

*2 Seropositive for both Pgp3 and CT694*

*3 Serology was not measured for a random sample of 6–9-year-olds at months 12 and 24*
