# Supplementary figures and images for: Predicting future community-level ocular Chlamydia trachomatis infection prevalence using serological, clinical, molecular, and geospatial data
Source: PLoS Negl Trop Dis. 2022 Mar 11;16(3):e0010273. doi: 10.1371/journal.pntd.0010273 (PMC8942265; doi:10.1371/journal.pntd.0010273)

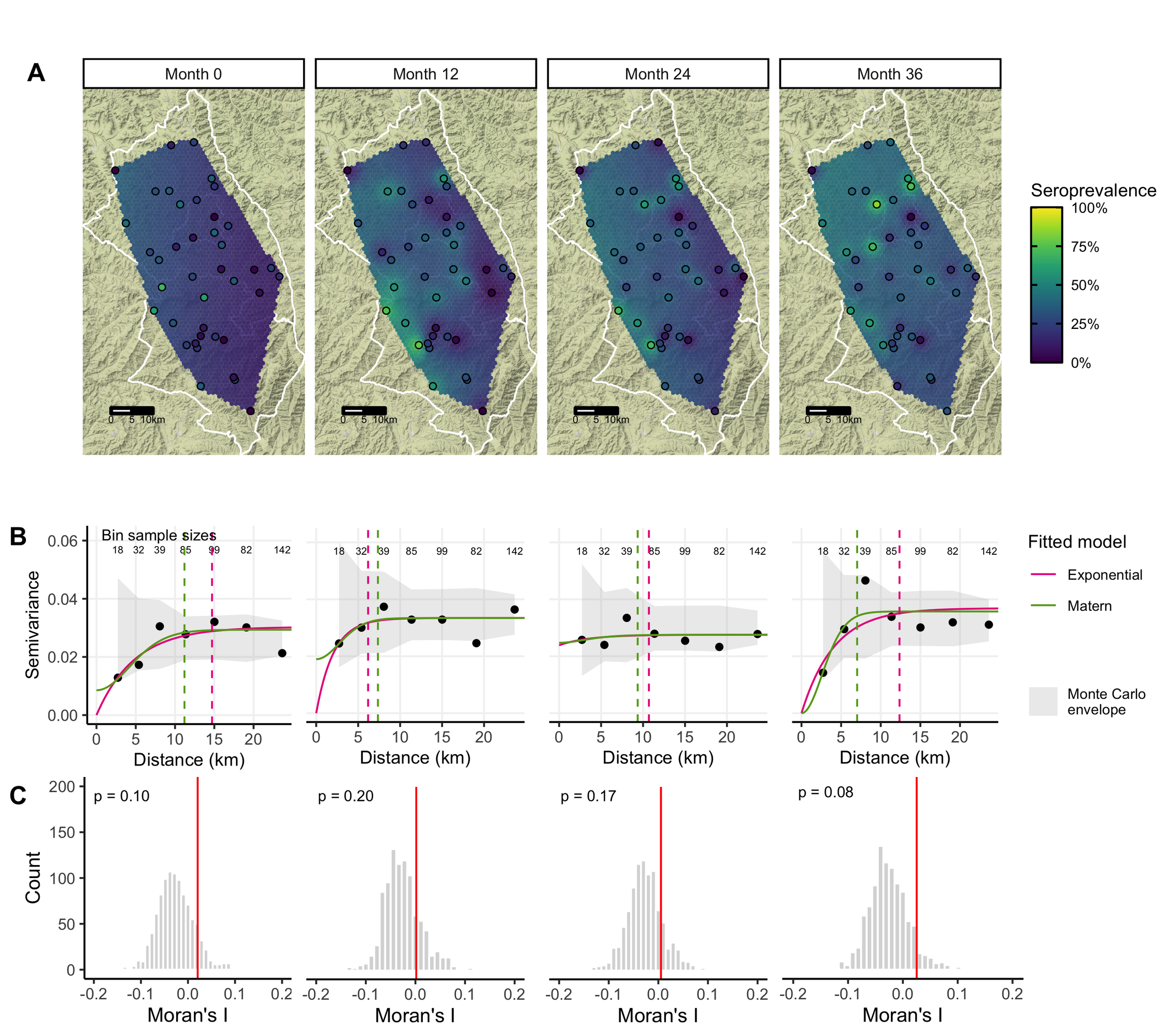

Supplement: S1 Fig — Maps (A), variograms (B), and Moran’s I (C) for seroprevalence among 0–5-year-olds at each study month. Maps display prevalence for 40 study communities at each follow-up visit, spatially interpolated over the convex hull using kriging. Variograms capture similarity between community-level prevalence measurements as a function of distance between community pairs (in km), with smaller semivariance values representing increased similarity. Exponential (magenta) and Matérn (green) models were fit to each empirical variogram, and the effective range (dashed vertical line) is defined as the distance at which the fitted model reaches 95% of the sill. The Monte Carlo envelope (gray shading) displays pointwise 95% coverage of 1000 permutations, representing a null distribution. Moran’s I was calculated over 1000 permutations (gray bars, with observed value represented by red line), and a permutation-based p-value was calculated. The base map layer for panel A in this figure was downloaded from Stamen Maps (“Terrain”) and is available under the CC BY 3.0 license. (TIF) [file pntd.0010273.s004.tif]

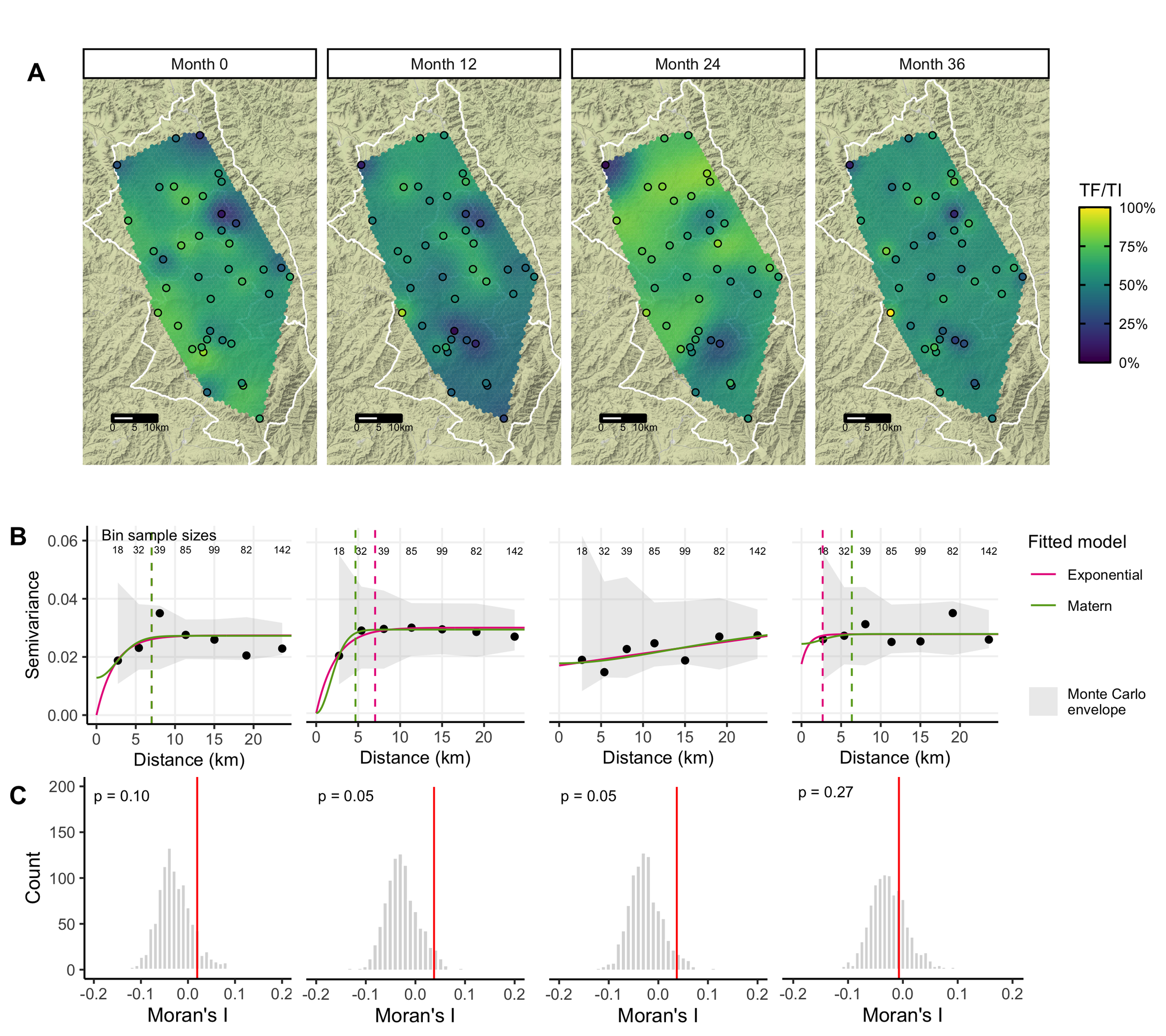

Supplement: S2 Fig — Maps (A), variograms (B), and Moran’s I (C) for active trachoma prevalence among 0–5-year-olds at each study month. Maps display prevalence for 40 study communities at each follow-up visit, spatially interpolated over the convex hull using kriging. Variograms capture similarity between community-level prevalence measurements as a function of distance between community pairs (in km), with smaller semivariance values representing increased similarity. Exponential (magenta) and Matérn (green) models were fit to each empirical variogram, and the effective range (dashed vertical line) is defined as the distance at which the fitted model reaches 95% of the sill. The Monte Carlo envelope (gray shading) displays pointwise 95% coverage of 1000 permutations, representing a null distribution. Moran’s I was calculated over 1000 permutations (gray bars, with observed value represented by red line), and a permutation-based p-value was calculated. The base map layer for panel A in this figure was downloaded from Stamen Maps (“Terrain”) and is available under the CC BY 3.0 license. (TIF) [file pntd.0010273.s005.tif]

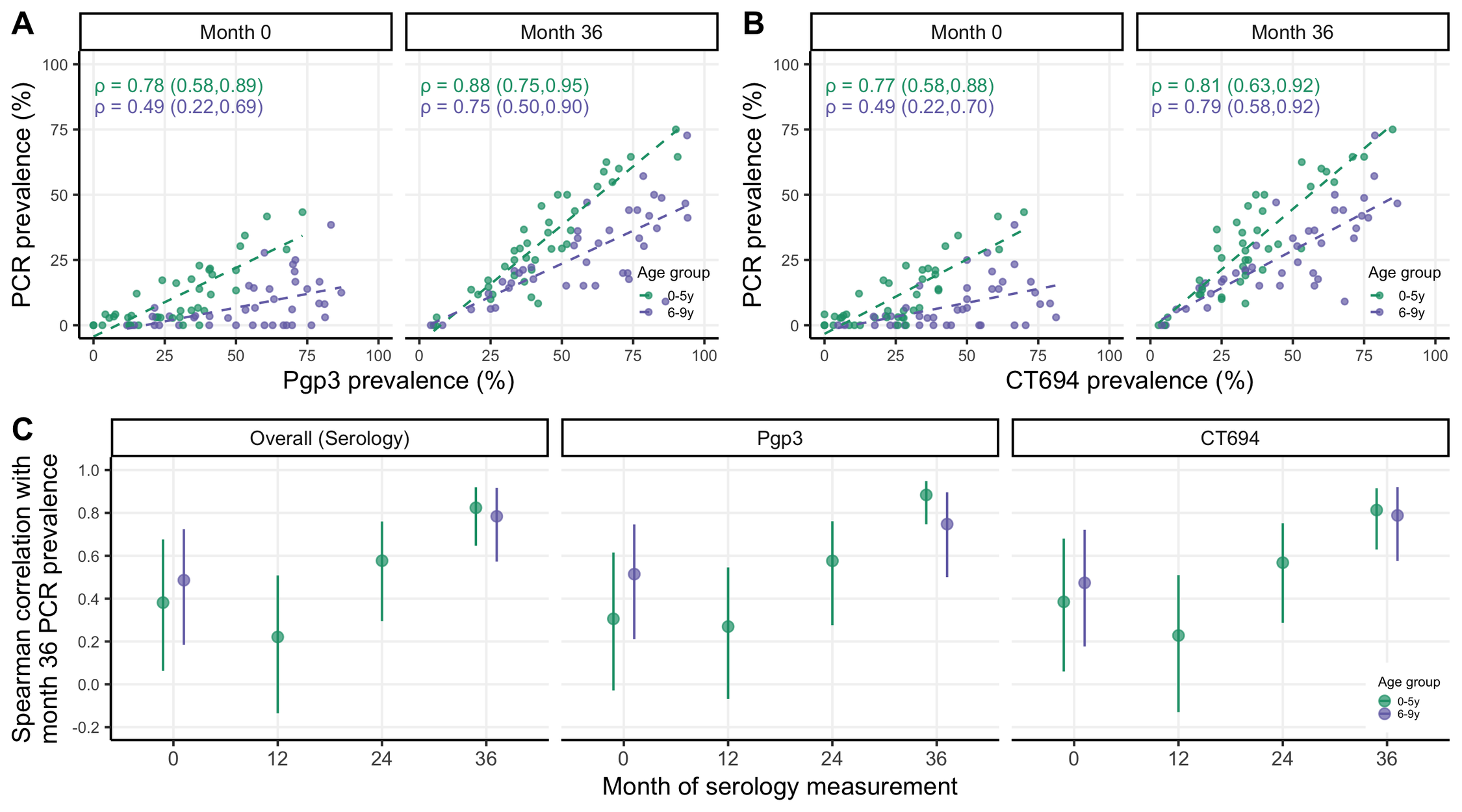

Supplement: S3 Fig — Panels display Spearman rank correlations between community-level Pgp3 seroprevalence and PCR prevalence at months 0 and 36 (A), CT694 seroprevalence and PCR prevalence at months 0 and 36 (B), and PCR prevalence at month 36 and seroprevalence measured at each follow-up visit across 40 study communities (C). Correlations are shown separately for 0–5-year-olds (green) and 6–9-year-olds (purple) when possible, and 95% confidence intervals were estimated from 1000 bootstrap samples. Serology data was not collected for a random sample of 6–9-year-olds at months 12 and 24. (TIF) [file pntd.0010273.s006.tif]

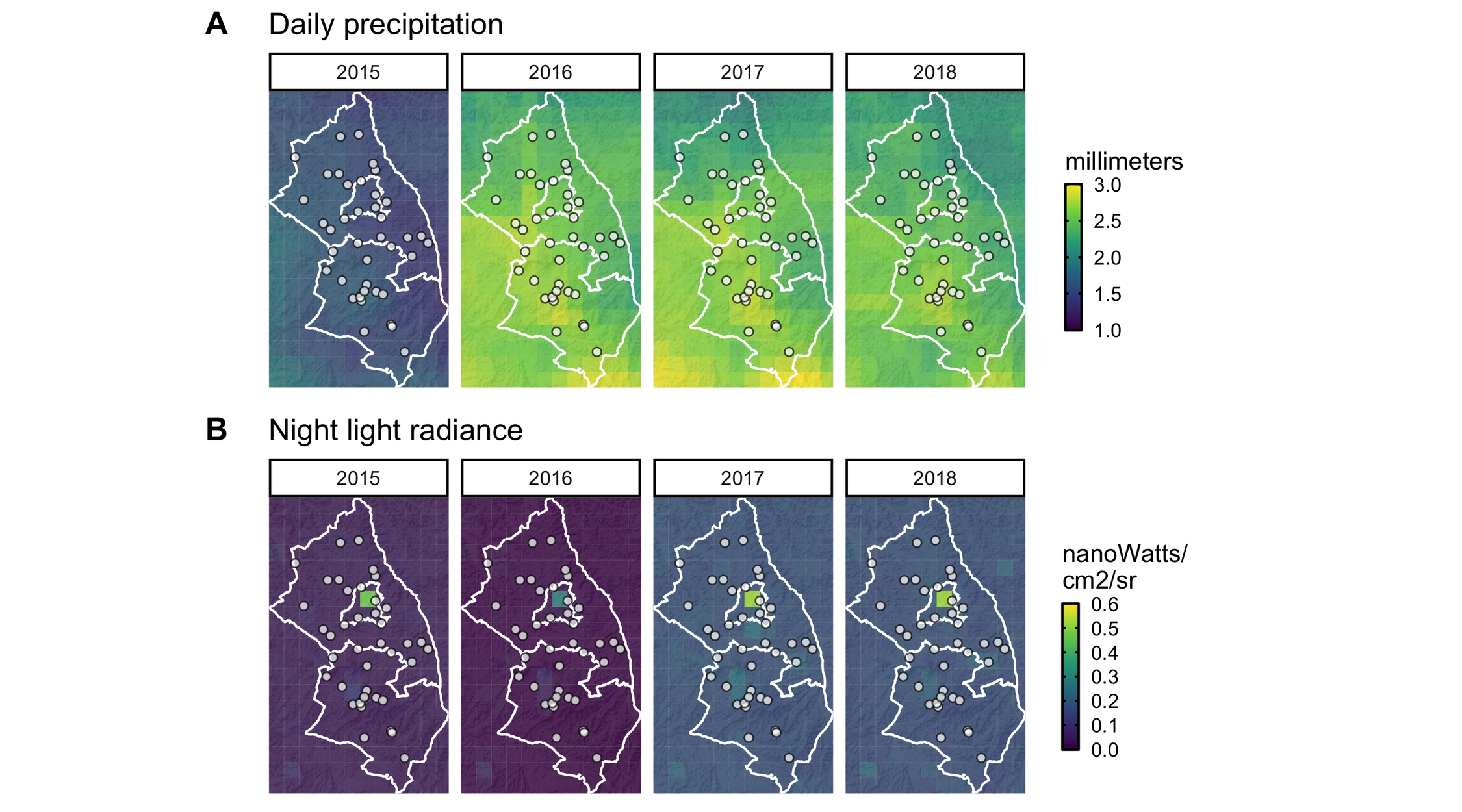

Supplement: S4 Fig — Variables were estimated for 240 grid cells of 2.5 x 2.5 arc minutes (approximately 20 km2 at the median latitude of the study area). Daily precipitation (A) and monthly night light radiance (B) averaged over the year were included in the final set of prediction models. The base map layer for this figure was downloaded from Stamen Maps (“Terrain”) and is available under the CC BY 3.0 license. (TIF) [file pntd.0010273.s007.tif]

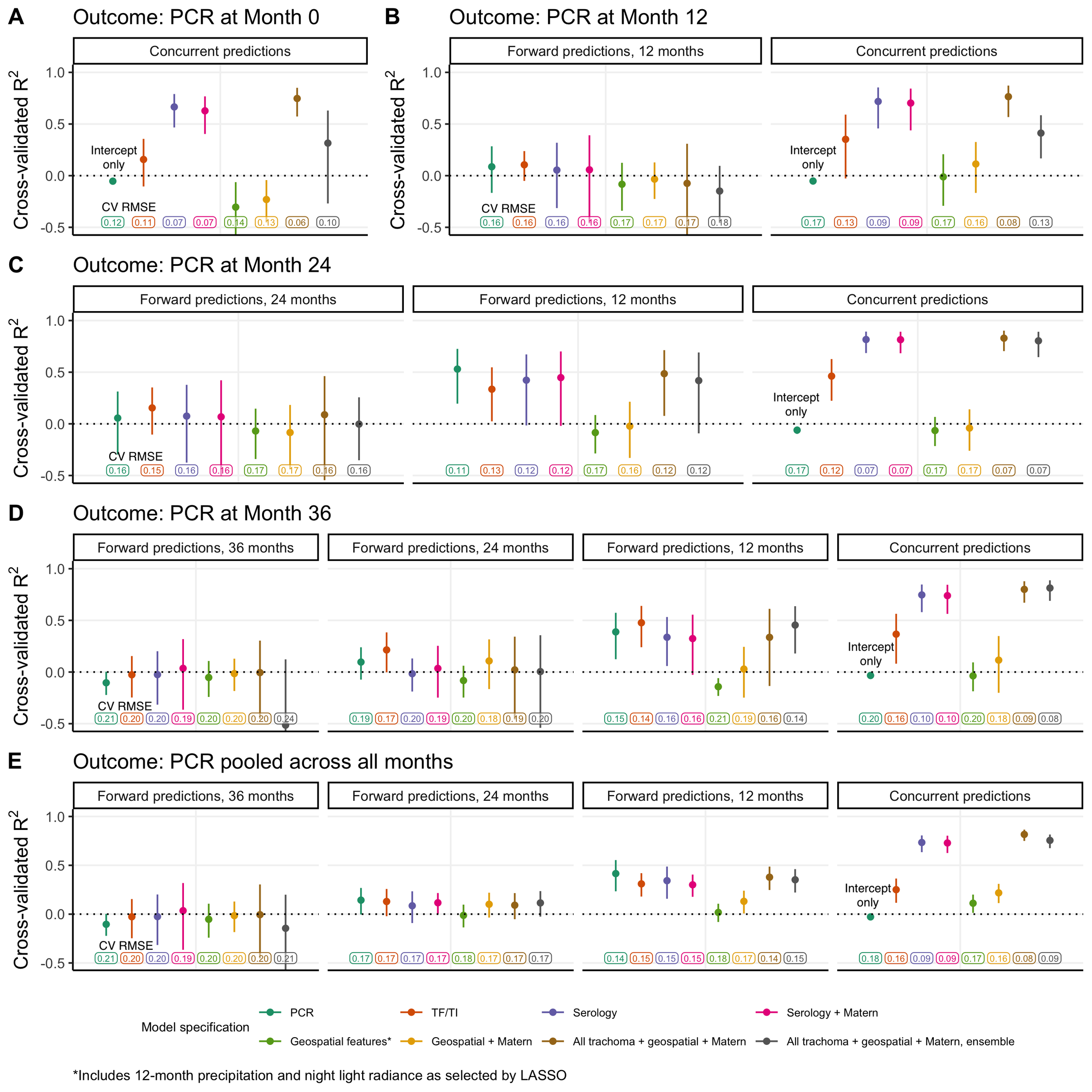

Supplement: S5 Fig — Cross-validated R2 for models predicting community-level PCR prevalence among 0–5-year-olds at month 0 (A), at month 12 (B), at month 24 (C), at month 36 (D), and pooled across all months (E). Cross-validated R2 (coefficient of determination), 95% influence-function-based confidence interval, and cross-validated root-mean-square error (RMSE, text label) are shown for each model specification. Blocks of size 15x15km were used for 10-fold spatial cross-validation. (D) is equivalent to Fig 4 in the main text and is included here for comparison. (TIF) [file pntd.0010273.s008.tif]

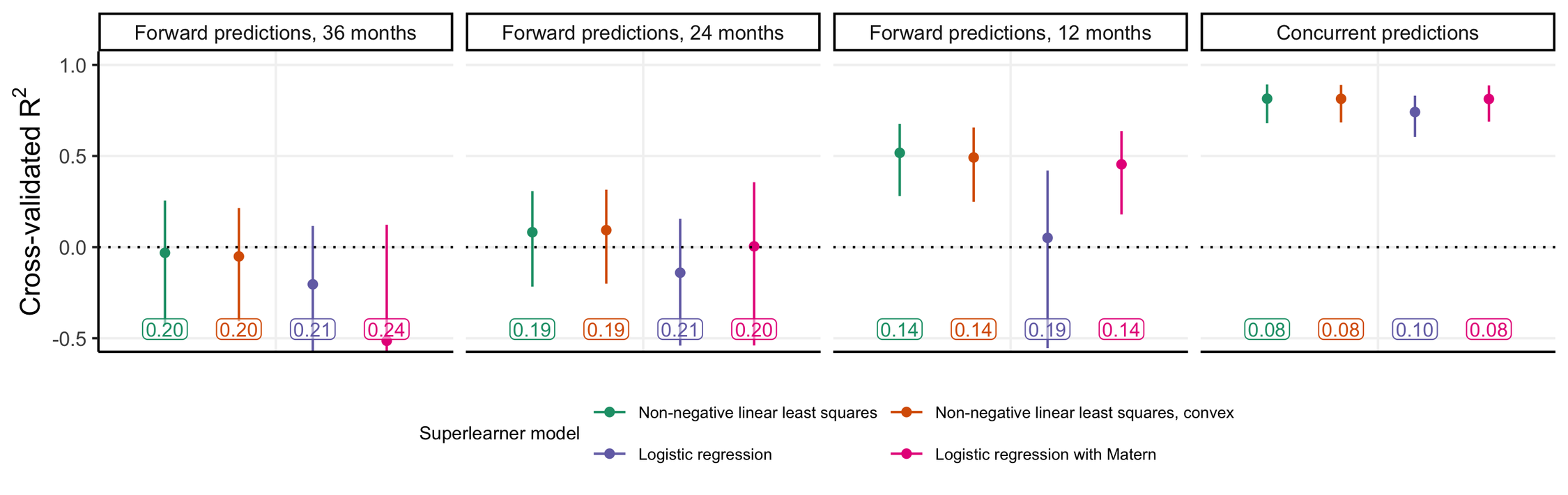

Supplement: S6 Fig — Cross-validated R2 (coefficient of determination), 95% influence-function-based confidence interval, and cross-validated root-mean-square error (RMSE, text label) are shown for each model specification. Blocks of size 15x15km were used for 10-fold spatial cross-validation. (TIF) [file pntd.0010273.s009.tif]

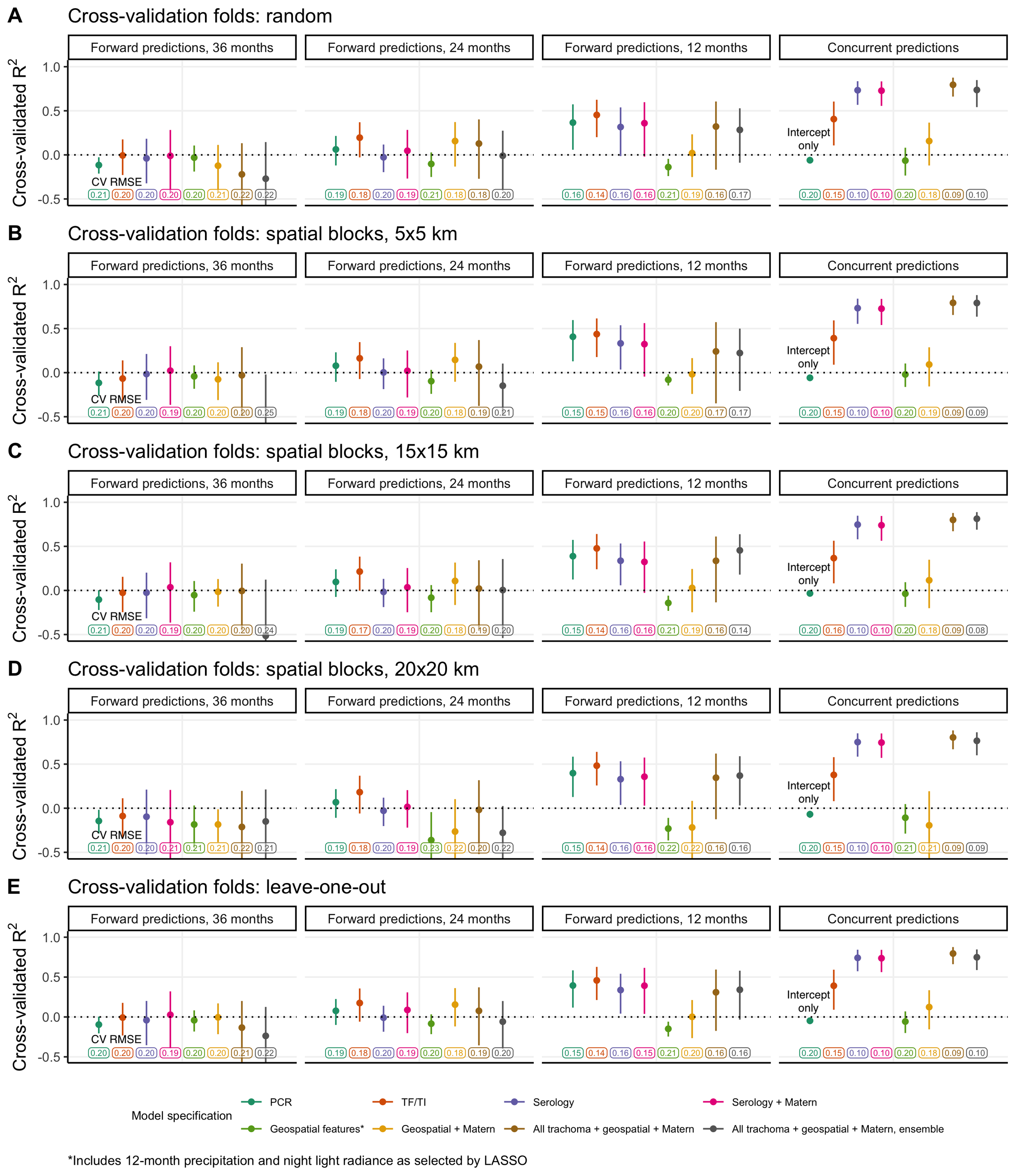

Supplement: S7 Fig — Cross-validated R2 for models predicting community-level PCR prevalence among 0–5-year-olds at month 36 using random 10-fold cross-validation (A), 10-fold spatial cross validation with 5x5 km blocks (B), 15x15 km blocks (C), and 20x20 km blocks (D), and leave-one-out cross-validation (E). Cross-validated R2 (coefficient of determination), 95% influence-function-based confidence interval, and cross-validated root-mean-square error (RMSE, text label) are shown for each model specification. (C) is equivalent to Fig 4 in the main text and is included here for comparison. (TIF) [file pntd.0010273.s010.tif]

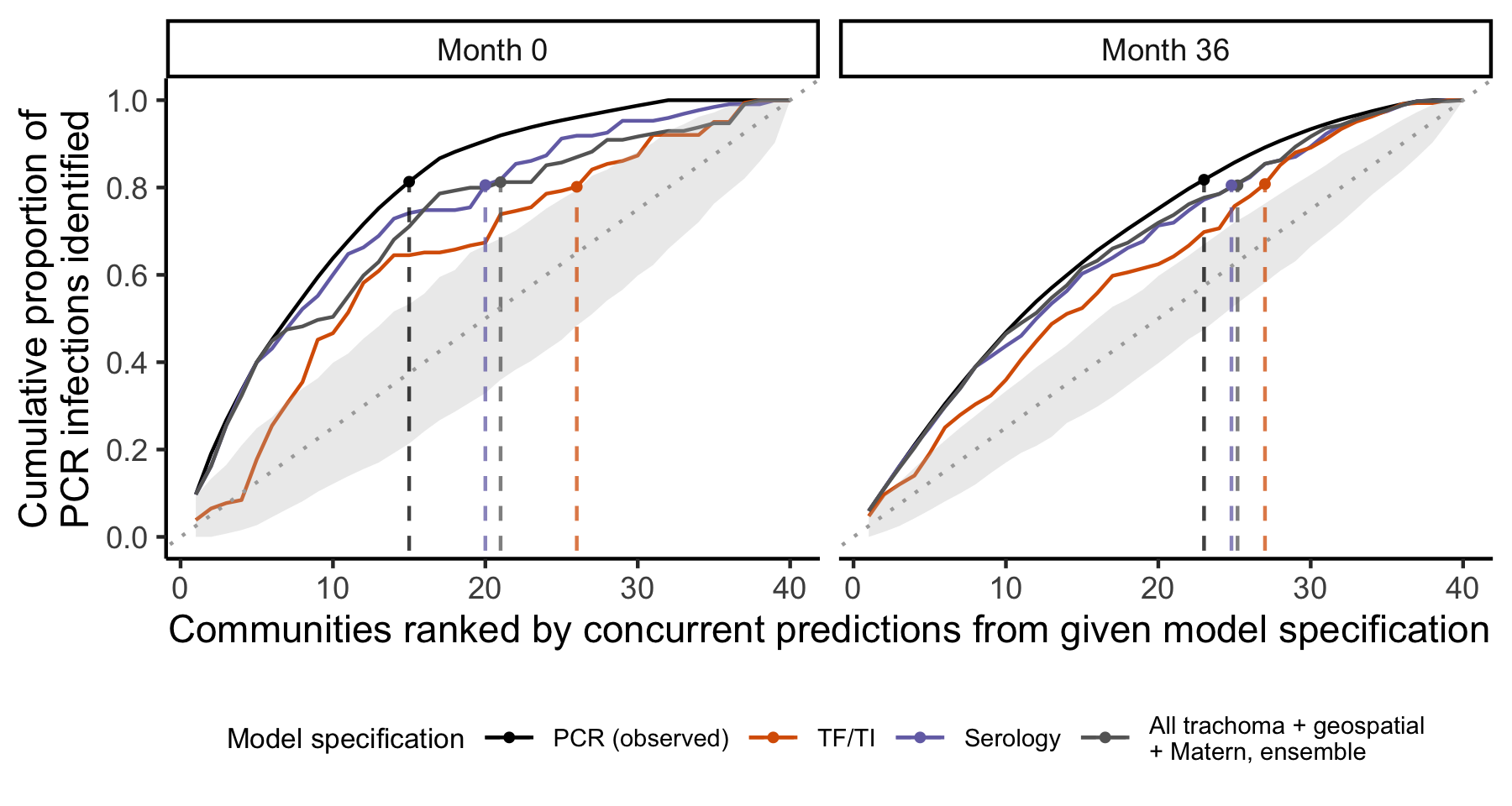

Supplement: S8 Fig — The black line in each facet represents the optimal ordering of scaled PCR infections at each respective month. Dashed lines indicate the point at which the cumulative proportion of infections, scaled to represent a sample of 30 individuals per community, surpassed 80%. To simulate a null distribution, we estimated the cumulative proportion of infections identified for 1000 random orderings of the 40 communities and plotted the 95% pointwise envelope (gray shading). At month 36, a model using only serology performed equally well to a model using all trachoma indicators, geospatial features, a Matérn covariance, and ensemble machine learning; vertical lines were offset slightly for visibility. (TIF) [file pntd.0010273.s011.tif]

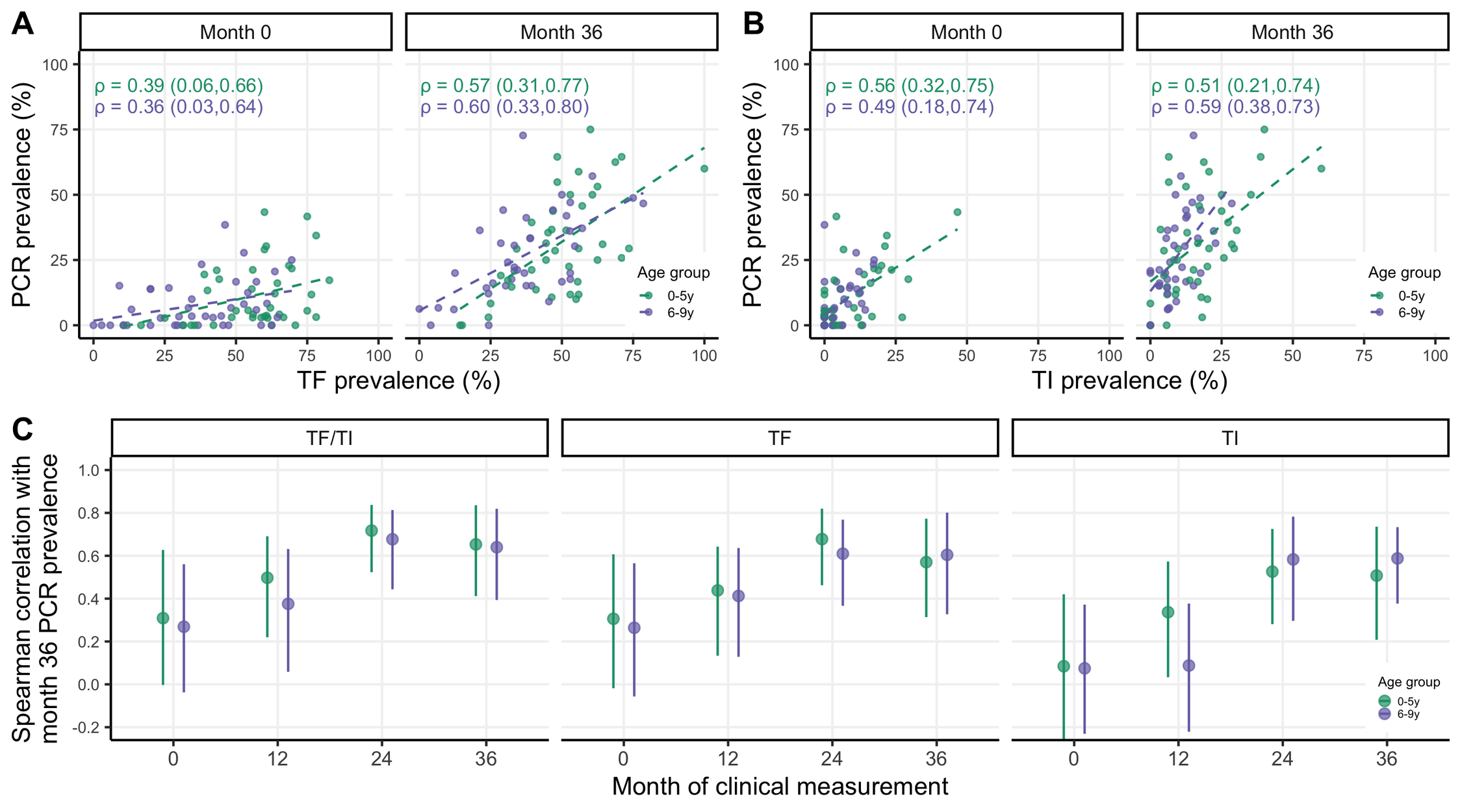

Supplement: S9 Fig — Panels display Spearman rank correlations between community-level TF prevalence and PCR prevalence at months 0 and 36 (A), TI prevalence and PCR prevalence at months 0 and 36 (B), and PCR prevalence at month 36 and active trachoma measured at each follow-up visit across 40 study communities (C). TF prevalence included any child diagnosed with TF, regardless of TI status, and vice versa. Correlations are shown separately for 0–5-year-olds (green) and 6–9-year-olds (purple), and 95% confidence intervals were estimated from 1000 bootstrap samples. (TIF) [file pntd.0010273.s012.tif]

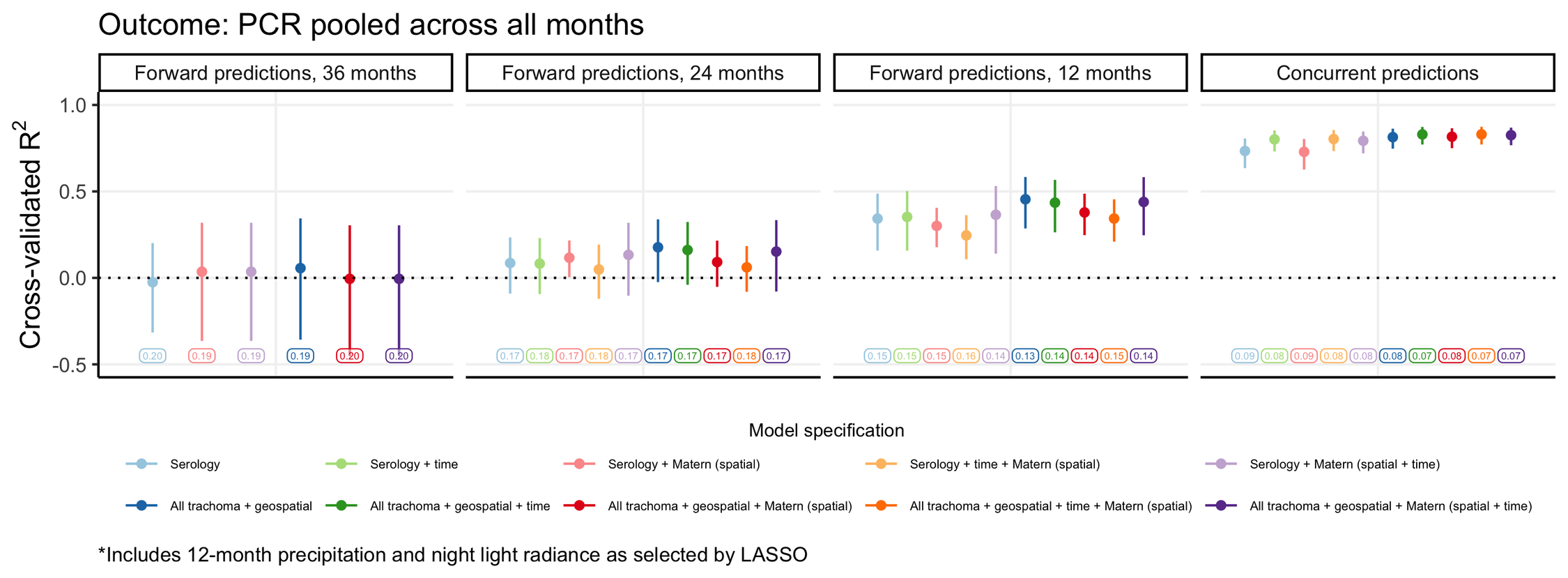

Supplement: S10 Fig — Cross-validated R2 (coefficient of determination), 95% influence-function-based confidence interval, and cross-validated root-mean-square error (RMSE, text label) are shown for each model specification. Blocks of size 15x15km were used for 10-fold spatial cross-validation. For predictions 36 months ahead, time could not be explicitly modeled as a linear covariate as all outcomes were measured at month 36. (TIF) [file pntd.0010273.s013.tif]
