# Supplementary material for: Predicting future community-level ocular Chlamydia trachomatis infection prevalence using serological, clinical, molecular, and geospatial data
Source: PLoS Negl Trop Dis. 2022 Mar 11;16(3):e0010273. doi: 10.1371/journal.pntd.0010273 (PMC8942265; doi:10.1371/journal.pntd.0010273)
Supplement: Attachment — Submitted filename: reviewer_responses.pdf [file pntd.0010273.s014.pdf]

Dear Reviewers and Editors,

Thank you for your thorough and insightful review of our manuscript. Your recommendations have helped to substantially improve and clarify this work, and we are looking forward to sharing this research with the broader scientific community. Please find below detailed responses to each of your comments, including line numbers referring to the clean updated manuscript.

Sincerely,
Christine Tedijanto, on behalf of all authors

Reviewer's Responses to Questions
Key Review Criteria Required for Acceptance?
As you describe the new analyses required for acceptance, please consider the following:

Methods
-Are the objectives of the study clearly articulated with a clear testable hypothesis stated?
-Is the study design appropriate to address the stated objectives?
-Is the population clearly described and appropriate for the hypothesis being tested?
-Is the sample size sufficient to ensure adequate power to address the hypothesis being tested?
-Were correct statistical analysis used to support conclusions?
-Are there concerns about ethical or regulatory requirements being met?

Reviewer #1: *Yes to all, though I do not profess deep expertise in geostatistical modelling*

Reviewer #2: *Ok*

Reviewer #3: *Methods: in general, the description of the methods section needs more detail to determine what was done.*

Line 108: only 40 communities in a zone were selected. How was the selection done? How many total communities exist in Wag Hemra zone? Please elaborate here. While spatial analyses can provide interpolation, assumptions if the data are too sparce become more problematic.

We have included additional information on community selection in the Methods as follows: “Communities were located in rural areas within a 4-hour drive and/or walk from the main road and included all households within 1.5 km of a potential water point (e.g. hand-dug well or protected spring) as determined by geohydrologic survey; further details are available in the study protocol” (lines 110-113).

We also expanded upon potential implications of this selection process in the Discussion: “...communities were purposely selected to be rural, but not too remote, and close to a potential

water point – as a result, our findings may not be generalizable to urban or extremely remote areas, and spatial interpolation across the study site should be interpreted cautiously" (lines 499-502).

Line 111: Please provide the dates of the trial here so that it can be placed in context with MDA.

Thank you for this helpful suggestion. We have added the dates of the trial (November 2015 through March 2019) at line 106.

Line 122: Please clarify for here and Line 257: Were 30 participants TOTAL from each community chosen, or 30 individuals in each age group, for a total of roughly 90 per community.

We apologize for any confusion this may have caused. We have clarified in both places that approximately 30 individuals in **each** age group were sampled, for a total of 90 per community (lines 123-124, 275).

Figure One. A map of all the communities, with the ones enrolled in the study in red, should be created. A visual picture of the potential for clustering would be ideal. This would also help us understand the paucity of villages selected in the south and southeastern areas of the study area if in general that area , which appears mountainous, is sparsely settled.

While we agree that a comprehensive map would be helpful for understanding the context of the selected communities, data to create this map are unfortunately not available to us. To help readers further understand potential implications of the selection process, we have provided additional information regarding selection of communities into the study (lines 110-113) and expanded upon selection (lines 499-502) and sparsity (lines 495-496) in the Discussion.

Line 125: Does molecular refer to a test for infection? Molecular tests are relatively nonspecific (for example, they are used for antibody testing as well) so perhaps using a term with more specificity would be useful or define its use here.

Yes, throughout this manuscript we used "molecular" to refer to PCR testing for infection. We have changed "molecular" to "PCR" at line 127 (previously 125) as recommended and at other key points in the text (e.g. line 175) for clarity.

Line 126: it is always tricky attempting to use clinical trial data for other purposes, and especially to combine data across arms. The authors state there was no difference in endpoint of community level ocular Ct infection among 0-5 year-olds between intervention arms-but is this based on a reduced sample size (since the age group six years and older is not included)? Are the point estimates very close as well? And what about the 6-9 year-olds? I would suggest they adjust for intervention arm as a variable, as I am not convinced yet that combining the data is across arms is justified.

This is an important point, and many of these concerns have been addressed in detail in the recently published primary outcomes paper for the RCT (https://www.thelancet.com/journals/langlo/article/PIIS2214-109X(21)00409-5). The pre-specified primary outcome of the study was ocular *Ct* infection among 0–5-year-olds, and this outcome is also reported among 6–9-year-olds in this paper. The main findings were as follows:

- After adjusting for baseline, the prevalence of ocular *Ct* infection did not differ between the two arms across the three post-randomization time points for 0–5-year-olds (risk difference 3.7 percentage points higher in the WASH arm, 95% CI: -4.9 to 12.4, p = 0.40, pre-specified primary analysis) or 6–9-year-olds (5.0 percentage points higher in the WASH arm, 95% CI: -2.0 to 12.1%, p = 0.16).
- At month 36, the mean prevalence of ocular chlamydia among 0–5-year-olds was 32% (95% CI: 24 to 41%) in the WASH arm and 31% (95% CI: 21 to 41%) in the control (delayed WASH) arm.

For the readers of this paper, we have provided additional information on the trial outcomes as follows: “At the end of WUHA, after adjusting for baseline, there was no statistically significant difference in the primary endpoint of community-level ocular Ct infection among 0–5-year-olds between intervention arms across the three post-baseline time points (risk difference: 3.7 percentage points higher in WASH arm, 95% CI: -4.9 to 12.4, p = 0.40)” (lines 127-131).

To address the concerns of the reviewer, we also tested our main analysis (Figure 4) with intervention arm as a covariate with very similar results:

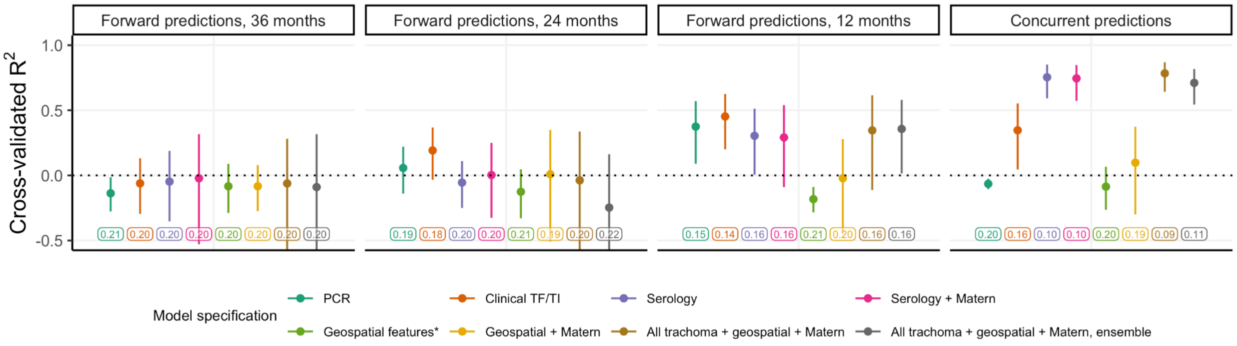

*Includes 12-month precipitation and night light radiance as selected by LASSO

Line 157: This reviewer is concerned that inferences on trachoma , infection, and serologic status are being made per community on the basis of 30 children, when it takes almost 2500 to have confidence in an estimate made in a district. For these correlations, how is the error of measurement in the underlying estimate of interest propagated into the correlation itself? And if not, why not?

Thank you for this insightful question. Although we have not explicitly propagated error of the underlying cluster-level estimates into the confidence intervals, we believe that our approach

sufficiently captures uncertainty in the correlation coefficients. We have added the following text to the Methods to explain our reasoning: "As communities were the unit of analysis, each bootstrap replicate consisted of forty communities resampled with replacement. This aligns with measures of uncertainty for cluster-level summaries which treat clusters as the primary source of variation" (lines 178-181).

In addition, the current surveillance strategy seeks to estimate a district-level mean by sampling 30-40 children from each of ~30-40 clusters (https://apps.who.int/iris/handle/10665/275523). Our analysis similarly estimates a population-level parameter (correlation) using approximately the same number of children and clusters.

Line 135: Please provide more information on the training of the graders. How many graders were involved, what was the training they received? Importantly, were the graders assigned to communities at random, or were they clustered in the woredas? If so, what was the agreement between them in grading?

We have added the following information regarding training: "Each year, eight local nurses and other healthcare professionals were recruited to serve as trachoma graders and swabbers. These individuals completed a four-day training with two days of classroom training and two field practice days. Prior to participation in fieldwork, graders were required to pass a photographic grading test with a Cohen's kappa score of 0.6 or greater relative to consensus grades from a panel of three expert graders. Grading teams were randomly assigned to clusters" (lines 137-142).

Line 140: Were air swabs taken to control for contamination in the field/lab? If so, what were the findings? Please provide the criteria by which pools were selected for unpooling, including the "other characteristics" and how often this occurred.

We have specified details about air swabs and the criteria for unpooling as follows: "Swabs from positive pools were tested individually for 0–5-year-olds at all visits, for 6–9-year-olds at months 12, 24, and 36, and if >80% of pools for a cluster were positive for all other age groups and time points. Approximately 12% of samples from 6–9-year-olds with an equivocal or positive pooled result at baseline were also tested individually. Air swabs were collected in every cluster at the beginning and end of each monitoring visit. None of the air swabs tested positive for *Ct*" (lines 157-162).

Line 159: Apparently predictive correlations were done based just on the 0-5 year-olds, and this should be stated here.

We agree that this should be clearly stated and have begun the section entitled "Predictive model selection" with the following: "Prediction models were limited to children 0–5 years old…" (line 197).

Somewhere in the methods, please provide a sample size estimate or at least a power calculation given the number of children per community and 40 communities.

We have added the following to the Methods: "As this was a secondary analysis, the sample size was fixed at 40 communities per survey. To our knowledge, there are no methods available to estimate power for cross-validated error in prediction problems. Instead, we estimated the minimum detectable effect for the correlation analysis. Assuming a two-tailed alpha of 0.05, we had 80% power to detect a correlation of 0.43 or larger with 40 communities" (lines 262-266).

<u>Results</u>
-Does the analysis presented match the analysis plan?
-Are the results clearly and completely presented?
-Are the figures (Tables, Images) of sufficient quality for clarity?

Reviewer #1: *Yes to all*

Reviewer #2: *Ok*

Reviewer #3:

Line 257. Please provide test of significance for the statement that seropositivity increased among 0-5 year-olds, because unless the test for trend is significant, I do not see a significant difference from baseline to 36 months.

We agree with the reviewer that this observation requires additional statistical support. Since the number of clusters in our data is relatively small, we evaluated whether there was a statistically significant difference between seroprevalence at baseline and at 36 months among 0–5-year-olds using both a non-parametric Wilcoxon signed-rank test and a linear mixed effects model with a random intercept for each study cluster. Both methods found a significant difference between seroprevalence at baseline and month 36 with two-sided p-values of 2.6×10^{-4} and 1.8×10^{-10}, respectively. We have reported results of the non-parametric test in the text at lines 282-283 and have also added a p-value for the same test among 6–9-year-olds indicating no statistically significant difference (line 284).

Figure 2b: shouldn't the title be Semivariogram, if the figure is the spatial dependence of the semivariance as stated?

"Variogram" and "semivariogram" are used interchangeably in the geostatistical literature for this function. As "semivariance" is somewhat of a misnomer (doi: 10.1007/s11004-011-9348-3), we chose to use the term "variogram" throughout this work.

Figure 3 and Figure 4. Perhaps I am reading this incorrectly, but it appears to me that clinical TF/TI at 24 months correlates better with PCR outcomes at 36 months than does serology or even PCR at 24 months. This is worth mentioning, because the readers will notice it and want to have the authors take on the finding. Although serology was more correlated in the concurrent measurements, if one is trying to determine who is at risk going forward, could clinical TF one year earlier provide clues? This is also worth mentioning in the discussion, and the authors thoughts on if the correlation is strong enough, correlation of 0.75, to be useful.

We agree that this is an interesting observation. We have noted that "[w]hen predicting 12 months into the future, all trachoma indicators performed moderately well" (line 353) rather than highlight clinical trachoma alone, due to the substantial overlap in cross-validated R^2 confidence intervals across models. For readers who may be curious about this, we have added the following to the Discussion (lines 465-469):

> "Interestingly, active trachoma maintained a fairly consistent and strong correlation (~0.7) with PCR prevalence at both 0 and 12 months into the future (Figure 3C) and was a slightly better predictor of PCR prevalence 12 and 24 months ahead compared to serology (Figure 4); however, these findings should be interpreted with caution due to substantial uncertainty in predictive performance across models and inconsistent trends in active trachoma (Table 1)."

Paragraph starts line 349 : I am concerned that in this setting of very high rates of infection, while high burden communities are truly in need of MDA, it does not mean the other 17 communities do not have worrisome infection. Please show the infection rates in the other 17 communities. By using this approach to identify high burden, what is the likelihood that we are missing communities for intervention that still have infection rates greater than 5%, for example? Would another approach that identifies the communities that have very low infection (efficient identification of low burden communities) be a better way to screen out communities?

We acknowledge the reviewer's concern that the "unidentified" villages may have worrisome levels of infection in this highly endemic context (median PCR prevalence: 14%, range: 0 to 25% among the 17 communities comprising 20% of *Ct* infections). However, our goal with this analysis was not to explicitly classify "high burden" or "low burden" communities, which would warrant a different approach – rather, our goal was to efficiently (i.e. in the fewest number) identify communities comprising the majority of infections over the entire study area (defined arbitrarily as 80% here). In other words, this analysis explores the ability of the predictive models to rank order communities rather than their ability to accurately predict prevalence.

This application is meant to be largely illustrative and would likely be most useful as infections concentrate in fewer communities (e.g. at baseline, see Figure S9). We have added further text to clarify this objective as follows: "We also illustrate a potential application of predictive models to rank order and therefore efficiently identify communities with high infection burden; we expect that this approach may be most useful when infections are concentrated in a small number of communities" (lines 416-419).

<u>Conclusions</u>

-Are the conclusions supported by the data presented?
-Are the limitations of analysis clearly described?
-Do the authors discuss how these data can be helpful to advance our understanding of the topic under study?
-Is public health relevance addressed?

Reviewer #1: *Yes to all*

Reviewer #2: *Ok*

Reviewer #3:

limitations section is needed, the public health relevance needs more discussion as outlined below

Line 466. Conclusions: this reviewer is concerned with a conclusion that serological markers may be well suited for community level monitoring, without a fuller discussion on the practical implementation of the suggestions. At least a discussion of the need to study cost and resource balance of undertaking monitoring at community level versus precision targeting of antibiotic is needed. At present, districts will need a sampling approach to monitor some communities at some time points, and at each time point always in a position of not including some communities, which has an unknown effect on outcomes. I recognize that this study was not a true district study and was not designed to test how to implement seroprevalence strategically in a district, but in fact given the authors conclusions, some attention to this issue is warranted.

Thank you for this thoughtful suggestion. In lieu of gathering all of the limitations into a specific section, we have opted to arrange the Discussion topically, with comparisons to existing literature paired with relevant limitations from this study (see lines 479-484, 493-496, 502-504). We agree with the reviewer that a more detailed discussion of public health relevance is warranted and have added the following text to the Discussion (lines 513-517):

> “Next steps towards programmatic implementation of serosurveillance for trachoma should focus on further development of survey design and analytic methods including model-based geostatistics, which has recently been applied to trachomatous trichiasis, cost-effectiveness analyses to weigh the benefits of targeted interventions against the costs of fine-scale monitoring, and consideration of integrated serosurveillance programs to enable scalability.”

Summary and General Comments
Use this section to provide overall comments, discuss strengths/weaknesses of the study, novelty, significance, general execution and scholarship. You may also include additional comments for the author, including concerns about dual publication, research ethics, or publication ethics. If requesting major revision, please articulate the new experiments that are needed.

Reviewer #1: *Trachoma is an important public health problem. This is an important addition to the literature on it. It's an elegant study and has been very well written-up. To provide some sense that I have done my job in providing peer review, I am forced to provide a list, below, of what amounts to extremely minor comments. But also: I am a pedant.*

Great paper, though. I thoroughly enjoyed reading it.

The authors are quite right to use as their outcome variable the prevalence of conjunctival C. trachomatis infection rather than, say, the prevalence of TF. Although programmes currently use the prevalence of TF in 1-9-year-olds as a measure of success, this reflects the fact that when targets for trachoma programmes were being defined, assays for C. trachomatis infection were felt to be either too expensive, too insensitive or too unavailable to be used to monitor progress. Decisions on whether or not to apply antibiotic MDA should absolutely be based on the presence or absence of significant levels of transmission of the organism targeted by the antibiotic in question.

Thank you for your helpful and clear comments!

Line 81: please change "prevalence above 5%" to prevalence ≥5%"

This change has been incorporated.

Line 122: please change "three age groups (0-5, 6-9, 10+)" to "three age groups (0-5 years, 6-9 years, 10+ years)"

This change has been incorporated.

Line 122: I think the authors mean "thirty individuals in each of three age groups…" Is that correct? If yes, suggest amend the sentence.

Yes, that is correct, and the sentence has been modified as suggested.

Line 124: suggest delete "old" since the word "aged" already appears before the numbers

This change has been incorporated.

Line 135: please change the "X" to a multiplication sign

This change has been incorporated.

Lines 136 and 137: on each line, please change the hyphens after "inflammation" to em-dashes, without flanking spaces, as recommended in the report of the 4th Global Scientific Meeting on Trachoma

This change has been incorporated.

Line 137-138: please change "An individual was considered positive for clinical trachoma if either TF or TI was detected" to "An individual was considered positive for active trachoma if either TF or TI was detected"

This change has been incorporated.

Line 216: suggest change one of the "also"s in this sentence to another word. For example, "We additionally explored…"

We have changed the first "also" to "additionally" as suggested.

Lines 253-255: The abbreviations TF and TI have already been defined, and "clinical disease" (by which the authors mean [active trachoma]: please see note above) has, too. The definitions of the signs TF and TI have not yet been given. It might be better to add this material to the section that is currently at lines 136-137. Please note that to be significant, follicles contributing to a diagnosis of TF have to (a) be in the central part of the upper tarsal conjunctival (not just "on the upper eyelid") and (b) be at least 0.5mm in diameter; to be significant, the inflammatory thickening of TI has to be "pronounced" such that more than half of the normal deep tarsal vessels are not visible because they are obscured by inflammatory infiltration. I think it is worth adding these details

Thank you for this recommendation. We have removed the prior definitions and added details about TF and TI diagnosis at lines 145-149 as follows: "Specifically, TF is characterized by the presence of five or more follicles which are (a) each at least 0.5 mm in diameter and (b) located in the central part of the upper tarsal conjunctiva. TI is distinguished by pronounced inflammatory thickening of the upper tarsal conjunctiva that obscures more than half of the normal deep tarsal vessels."

Lines 255, 270, 297-298, 302, 308, 324-325, 375-376, 413, 417, 422, 436, 443-444, abstract line 36: please change "clinical disease" or "clinical trachoma" to "active trachoma"

The terms "clinical disease" and "clinical trachoma" have been changed to "active trachoma" throughout the text, tables and figures.

Table 1: the word "clinical" in front of "TF/TI" in the column heads is redundant; please delete

We have removed “clinical” in front of “TF/TI” throughout the text, tables and figures.

Line 269: please change “active ocular infection” to just “ocular infection”, or even better, “conjunctival infection” (since only the conjunctiva was sampled). Adding the adjective “active” risks confusion between [active trachoma] and [infection], which is already too widespread. The word “active” should also be deleted from lines 378 and 383.

Thank you for this clarification; we have removed the word “active” but used the term “ocular infection” for consistency throughout the paper.

Line 296: can the authors please provide a little more numerical clarity to the expression “very likely”?

Thank you for this suggestion; we have specified as follows: “In a longitudinal cohort nested within the study, among 816 observations (representing 471 unique children) of consecutive serology measurements with an initial seropositive result, 91% remained seropositive one year later” (lines 321-324).

Line 312: suggest change “serology data was” to “serology data were”

This change has been incorporated.

Reviewer #2: *Well written and good focused study on Predicting future ocular Chlamydia trachomatis infection prevalence using serological, clinical, molecular, and geospatial data.*

Thank you!

Reviewer #3:

Title. The title should make clear that this is prediction at community level. Trachoma programs focus on district level surveillance, using sampling to represent the district. Identifying communities within districts is another level of complexity altogether.

This is a good point, thank you. We have edited the title to read “Predicting future **community-level** ocular *Chlamydia trachomatis* infection prevalence using serological, clinical, molecular, and geospatial data.”

Abstract

Line 31: Please indicate if the communities were originally chosen at random or not. If not, the ramifications of this need to be addressed in the discussion.

Thank you for raising this important point. The communities were not selected at random. As mentioned above, we have expanded on details of selection in the Methods (lines 110-113) and have added several lines regarding the impact of this to the Discussion (lines 499-502).

Line 33. The baseline findings need to be placed in context by indicating that MDA occurred 6 months prior to baseline. Infection would be much lower due to MDA, but disease burden would not yet have declined, as it does by month 12 in both age groups. I would suggest not devoting too much abstract space to baseline findings but rather focus on 24-month associations and then the concurrent associations.

As suggested, we have incorporated MDA into the Abstract as follows: "Median *Ct* infection prevalence among children 0-5 years old increased from 6% at enrollment, in the context of recent mass drug administration (MDA), to 29% by month 36, following three years without MDA" (lines 31-33). However, we have maintained baseline findings in the Abstract to highlight that, in this setting, serology continued to be a strong correlate of community-level infection prevalence even in the presence of MDA. This observation is a key finding of our work, especially in contrast with the relationship between infections and active trachoma (TF/TI) which was weaker in the context of MDA both in this analysis and in existing literature.

Line 42: the conclusion that serologic markers may be a programmatic tool has not been shown by this study, unless the authors discuss or theorize how this might be implemented in a programmatic setting. The evaluation of this tool in this study was done in a sample of communities, but how it might be extended to manage detection in communities not sampled is unclear.

We have removed the word "programmatic" from line 42 (now line 41) in the Abstract.

Introduction

Line 76: The authors nicely describe the evidence of trachoma clusters at village level, and high burden villages that are not apparent in overall EU estimates have been shown in previous studies. However, to argue that fine scale estimates of trachoma at community level could target limited resources is to ignore the fact that significant resources would be needed to undertake these estimates before targeting allocation of resources to communities with the highest burden. This issue needs to be addressed in the discussion, as a nuance to the rationale as stated here.

We agree with the reviewer that a more detailed discussion of public health relevance, including potential costs and benefits of this approach, is warranted and have added this to the Discussion at lines 513-517 as mentioned in an earlier response.

Discussion

The discussion section is balanced and well written. It would benefit from more discussion as suggested in the comments above. In addition, a discussion would be helpful of why the 6-9

year-olds may not have been as informative in a high prevalence setting (they already had high seropositivity to begin with, and the increase in infection was more modest in this age range).

We have expanded upon potential reasons that 6–9-year-olds may have been less informative in this setting in the Discussion: “The lower correlation among 6–9-year-olds is likely due to the discordance between dampened transmission due to MDA and high seroprevalence from past exposures in this older age group” (lines 458-460).

Arguments bolstering the use of younger ages would be enhanced by citing a paper by Odonkor et al (Plos NTD 2021) where re-emergence was clearly evident from strategic serostatus determination in the youngest children, who should have had no exposure to infection if the program has lowered transmission.

Thank you for recommending this relevant study. We have added a sentence and citation as follows: “...seroprevalence among younger children likely reflects more recent transmission patterns, an observation which has been leveraged to investigate potential recrudescence in Tanzania” (lines 460-462).

A section on limitations would also be reasonable to add, particularly the issues with small numbers of communities.

In order to provide readers with context for each of the key limitations of this work, we have integrated them throughout the Discussion. We have discussed the limitation of the small number of communities in our study at lines 495-496.
