# Supplementary material for: Predicting future community-level ocular Chlamydia trachomatis infection prevalence using serological, clinical, molecular, and geospatial data
Source: PLoS Negl Trop Dis. 2022 Mar 11;16(3):e0010273. doi: 10.1371/journal.pntd.0010273 (PMC8942265; doi:10.1371/journal.pntd.0010273)
Supplement: Attachment — Submitted filename: reviewer_responses.pdf [file pntd.0010273.s015.pdf]

Dear Reviewers and Editors,

Thank you for your comments on our updated manuscript! We appreciate your thorough review and have made edits as suggested. Please find below our responses to each of your recommendations.

Sincerely,
Christine Tedijanto, on behalf of all authors

Reviewer's Responses to Questions
Key Review Criteria Required for Acceptance?
As you describe the new analyses required for acceptance, please consider the following:

Methods
-Are the objectives of the study clearly articulated with a clear testable hypothesis stated?
-Is the study design appropriate to address the stated objectives?
-Is the population clearly described and appropriate for the hypothesis being tested?
-Is the sample size sufficient to ensure adequate power to address the hypothesis being tested?
-Were correct statistical analysis used to support conclusions?
-Are there concerns about ethical or regulatory requirements being met?

Reviewer #1: *Yes, yes, yes, yes, yes; no concerns about ethical or regulatory requirements*

Reviewer #2: *none*

Reviewer #3: *thank you for the detailed response, the changes have improved the clarity greatly. Please insert in Line 110 (of marked up manuscript) that the "communities were not selected at random. They were....". This makes it clear these are not a random sample and the readers do not have to infer.*

We have added this clarifying statement as suggested.

Results
-Does the analysis presented match the analysis plan?
-Are the results clearly and completely presented?
-Are the figures (Tables, Images) of sufficient quality for clarity?

Reviewer #1: *Yes, yes, yes*

Reviewer #2: *none*

Reviewer #3: *Line 322-325 of marked up manuscript. The addition of the cohort study is new, and not described in the methods. Cohort studies are quite complex and previous work shows antibody seropositivity stability is highly correlated with age even in the under five year olds. Unless there is a description (other than in the title of the figure) of how these children were selected at random, an analysis of the age distribution of the seropositives at baseline, how many were followed longitudinally, etc, these data should not be included as they are difficult to interpret. They add little to the manuscript and should be deleted along with figure S4.*

Thank you for this recommendation; we have removed this piece from the manuscript and supporting information.

Conclusions

-Are the conclusions supported by the data presented?
-Are the limitations of analysis clearly described?
-Do the authors discuss how these data can be helpful to advance our understanding of the topic under study?
-Is public health relevance addressed?

Reviewer #1: *Yes, yes, yes, yes*

Reviewer #2: *none*

Reviewer #3: *Very nice job with a full description of limitations*

Editorial and Data Presentation Modifications?

Use this section for editorial suggestions as well as relatively minor modifications of existing data that would enhance clarity. If the only modifications needed are minor and/or editorial, you may wish to recommend “Minor Revision” or “Accept”.

Reviewer #1: *The response to reviewer 3's comment about line 157 ("This reviewer is concerned that inferences on trachoma , infection, and serologic status are being made per community on the basis of 30 children, when it takes almost 2500 to have confidence in an estimate made in a district. For these correlations, how is the error of measurement in the underlying estimate of interest propagated into the correlation itself? And if not, why not?") could perhaps have taken into account the fact that surveys supported by GTMP/Tropical Data have, primarily for logistical reasons, almost always used compact segment sampling as the mechanism for selecting households and individuals within selected villages.*

That has the very helpful secondary advantage of meaning that cluster-level disease proportions are true cluster-level prevalences, assuming low levels of absenteeism, refusal and misdiagnosis. This point could perhaps be included in the discussion if the authors wished to do so, since it means that the methods that they propose could have even more applicability in the general case than in the specific dataset employed here (since the latter used a different sampling strategy at village level).

Thank you for this helpful comment. We have added the following text to the Discussion: "The methods used here may be extended to other surveys in which trachoma prevalence can be estimated at the cluster level, including those supported by the Global Trachoma Mapping Project and Tropical Data service which have typically relied on multi-stage sampling strategies with compact segment sampling within villages [added citations for Solomon et al. *Community Eye Health* 2019 and WHO Design parameters for population-based trachoma prevalence surveys 2018]" (lines 511-514).

Reviewer #2: *none*

Reviewer #3: *as noted above in two comments*

Summary and General Comments

Use this section to provide overall comments, discuss strengths/weaknesses of the study, novelty, significance, general execution and scholarship. You may also include additional comments for the author, including concerns about dual publication, research ethics, or publication ethics. If requesting major revision, please articulate the new experiments that are needed.

Reviewer #1: *I am satisfied with the changes that the authors have made to the paper in response to the first round of review.*

Reviewer #2: *well wirtten good focused following revision.*

Reviewer #3: *Overall, excellent revision and response to reviewers. Addition of one sentence and deletion of non-essential few lines as noted above are final comments.*
